# A Hybrid Motion Estimation for Video Stabilization Based on an IMU Sensor

**DOI:** 10.3390/s18082708

**Published:** 2018-08-17

**Authors:** Jutamanee Auysakul, He Xu, Vishwanath Pooneeth

**Affiliations:** College of Mechanical and Electrical Engineering, Harbin Engineering University, Harbin 150001, China; railway_dragon@163.com (H.X.); vpooneeth@ymail.com (V.P.)

**Keywords:** video stabilization, KLT tracker, motion estimation, IMU-aided stabilization, multi-threaded approach

## Abstract

Recorded video data must be clear for accuracy and faster analysis during post-processing, which often requires video stabilization systems to remove undesired motion. In this paper, we proposed a hybrid method to estimate the motion and to stabilize videos by the switching function. This method switched the estimated motion between a Kanade–Lucus–Tomasi (KLT) tracker and an IMU-aided motion estimator. It facilitated the best function to stabilize the video in real-time as those methods had numerous advantages in estimating the motion. To achieve this, we used a KLT tracker to correct the motion for low rotations and an IMU-aided motion estimator for high rotation, owing to the poor performance of the KLT tracker during larger movements. Furthermore, a Kalman filter was used to remove the undesired motion and hence smoothen the trajectory. To increase the frame rate, a multi-threaded approach was applied to execute the algorithm in the array. Irrespective of the situations exposed to the experimental results of the moving camera from five video sequences revealed that the proposed algorithm stabilized the video efficiently.

## 1. Introduction

Video stabilization is commonly used in unmanned aerial vehicles (UAVs), humanoid robots, binocular robot and so on, for surveillance, navigation, guidance, and control of the system through video data [1]. The jitter and burling effect in videos are mainly caused from the movement of the camera, also called the global motion, and due to the motion of the moving object in the existing video, which is termed the local motion. It requires reducing those phenomena for high-quality video output while the camera is in motion thus enabling usage of the other features, e.g., tracking, mapping and recognizing. This research is divided into analyzing the mechanical stabilization systems and the digital stabilization systems [2].

Firstly, the mechanical stabilization systems improved the support base of the camera by detecting the acceleration and angular velocity while the camera is moving [2]. In the camera market, the Optical Image Stabilization (OIS) system is installed on the camera lens or the image sensor which is quite expensive [3]. On the other hand, digital stabilization systems deal with image post-processing by compensating the movement of the captured image when the camera is moving. It can be divided into three steps, namely motion estimation, motion smoothing and image warping [4,5]. Motion estimation adopts the motion model, e.g., translation, affine, and similarity while two frames of the image source change the motion. Then, smoothening of the camera intentional motion is done by employing the Kalman filter [6] or a Gaussian low-pass filter [7,8] in order to eliminate the unplanned motion. Lastly, the stabilized video is warped on the final image plane.

The common technique for motion estimation in digital video stabilization is using block matching [9], a KLT (Kanade–Lucus–Tomasi) tracker [10,11], SIFT [12] and SURF [13], respectively. Some of the feature trackers such as SIFT and SURF have a heavy computational load for real-time digital video stabilization [14]. However, recently, Dong et al. [14] and Lim et al. [15] performed a KLT tracker to estimate the motion in real-time with a high frame rate and a low computational cost. A KLT tracker detected the feature points by Good Feature to Track and estimated the optical flow of consecutive frames with the Lucas–Kanade method. This tracker features success in evaluating the motion in the small movement but fails when exposed to a significant change in both local motion and global motion [15]. However, the large motion can be approximated by using IMU (Inertial Measurement Units) data as shown in [16] that demonstrates the efficiency of the gyroscope in estimating the optical flow during fast rotation. It adopted only the gyroscope data to aid the optical flow computation in improving the performance while measuring the motion estimations.

From the literature review, the motion distribution of global and local motion is limited to approximating the motion with only one motion estimation method. Therefore, we propose a hybrid function to switch the motion estimation algorithm to determine a transformation between two consecutive frames for stabilizing the video in the real environment. In case of low movement, we apply a KLT tracker to compute the optical flow of the moving object on two consecutive frames. However, in the case of fast rotation, the rotational data from an IMU sensor is used to estimate the movement by calculating the motion from the predefined moving point and the reference point. Thus, motion flow in each method can estimate the motion with rigid transformation. We then reduce the noise of motion by a Kalman filter which smoothens the trajectory. Finally, the stabilized video is warped.

The paper is organized as follows. In Section 2, we present a related work in video stabilization. In Section 3, we introduce our proposed framework concerning the methodology about the video stabilization using both a hybrid method and calibrated sensors. Next, we discuss a hybrid method in Section 4, which estimates the motion by swapping between a KLT tracker and an IMU-aided motion flow, and the homography approximation of the subsequence frames. Then, the motion filter used to reduce noise is explained in Section 5. Section 6 discusses the multi-threaded approach. Moreover, the efficiency of a hybrid method and the performance of the multi-threaded approach are presented in Section 7. Finally, the conclusions are provided in the last section.

## 2. Related Work

IMU sensors are used to estimate the motion of moving objects. To increase the motion tracking performance, an IMU sensor is also an integral part along with other types of sensors such as Zhao et al. [17] who used an GPS/INS (inertial navigation system) system to correct positions when compared with the standalone INS; Gadeke at al. [18] proposed an IMU sensor fusion with a barometric sensor for tracking the position of smartphones in case of lost connection; Carlos A. et al. [19] presented the motion tracking from human by using an IMU sensor, and a laser rangefinder to interact with robots; and Lake et al. [20] established the interface device between an IMU sensor and EMG sensor to detect the motion from muscles. Moreover, an IMU sensor was successful in tracking accurately the movement by combining an accelerometer and a gyroscope, as shown in [21,22]. However, our work focuses on only the gyroscope data of an IMU sensor used to measure the camera rotation for basic stabilization of videos.

In video stabilization applications, an IMU sensor is used in the mechanical system to reduce the jitter effect in devices, as demonstrated by Antonello et al. [23] who installed an IMU on the pan-tilt-zoom (PTZ) camera and used a two-level cascade to control a pneumatic hexapod’s support base. On the other hand, the IMU sensor in the digital video stabilization system is used only by the camera to estimate motion. Odelga et al. [24] successfully stabilized a video with a low computation load by using the orientation data from the support base relative to a horizontal frame with no feature tracking applied in the design. Drahansky et al. [25] used an accelerometer to estimate a local motion vector for calculating a smooth value in a global motion vector, while Karpenko et al. [3] used only gyroscope data to create rolling shutter wrapping for video stabilization. Additionally, other researchers have integrated IMU data and feature tracking. Moreover, some researchers have included both IMU data and feature tracking, respectively, e.g., Ryu at al. [26] who proposed the motion estimation by incorporating the rotation motion into the KLT tracking, which used the initial position from an IMU to predict the next frame on the robot’s eye application, thereby demonstrating speed and accuracy. Moreover, in the real-time of the video stabilization, Lim et al. [15] proposed a multi-threaded approach to improve the processing speed compared to Dong et al. [27], who used a notebook computer with a 2.5 GHz Intel Duo Core at 40 fps. However, Lim et al.’s method had an average frame rate at 50 fps while processing on a notebook computer with a dual-core 1.70 GHz processor.

## 3. Proposed Framework

**Figure 1:**
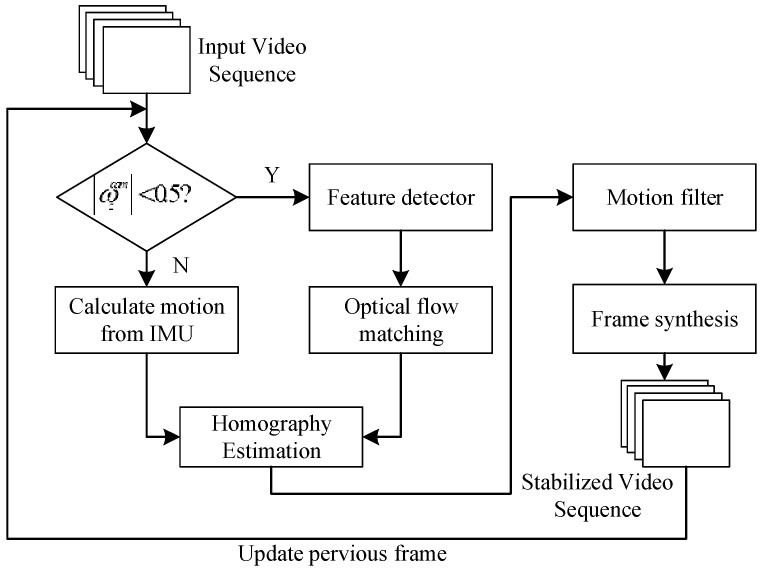
Flowchart of our proposed method.

The challenge in this paper is how to estimate the motion of the consecutive frames when using on the moving camera, e.g., the large rotates due to the camera rotations and the local movements based on the moving object in the scene. According to our objective, we applied a hybrid method to approximately decide the method to estimate the motion of the moving camera as illustrated by the flowchart in Figure 1.

Firstly, the rotational velocity of the camera needs to check for decision the function to estimate motion parameters between a KLT tracker and an IMU-aided motion estimator. Then, the motion estimation forward to approximate the homography by using the rigid transformation to warp the stabilized frame. However, the undesired motion may contain from the previous step then it can reduce the motion noise by the motion filter. In this paper, we use Kalman filter which suitable to the dynamic model. Lastly, the final stabilized frame is creating with the accurately affine matrix.

To estimate the motion with a hybrid method, we adopted the rotational velocity of the camera (*ω^cam^*) measured by an IMU sensor (*ω^imu^*) to decide the estimation method. However, the two devices were different positions thus we needed to transform *ω^imu^* to *ω^cam^* with the relative orientation (*R^ci^*). Hence, the rotational velocity of the camera is defined by:

(1) $$\omega^{cam}(t+t_{off})=R^{ci}\left(\omega^{imu}(t)+b^{imu}\right)$$

where *b^imu^* is the gyroscope bias, *t* and *t_off_* represent the measurement data from an IMU sensor in real-time and the temporal time offset, respectively. The camera and the IMU sensor are calibrated as follows: (1) calibrate the camera to find out the focal length (*f*) by using the camera calibration module in OpenCV (Open Source Computer Vision); (2) calibrate the gyroscope to prevent gyro drift problems with averaging of the bias offset. We can assess the bias offset by measuring the output signal of the gyroscope over a long period of time when this sensor is sitting still and reduce noise by Kalman filter; (3) estimate *R^ci^* by using the relation between gyroscope data and optical flow that was proposed by Li and Ren [16] which is using the CC+LS13 method, and (4) determine the *t_off_* between the gyroscope and the camera input by a cross-correlation (CC) method [28] to synchronize operation between both of the sensors to accurately collect data in time. Then, we can correctly estimate the *ω^cam^*.

The values of *ω^cam^* can be divided into two assumptions at 0.5 rad/s on the *z*-axis as IMU sensor values diminish in the low rotational rate and it continues to stabilize the video in this case by using a KLT tracker for the local motion in an existing video. Thus, both methods of motion estimation can be described in detail as the following subsection.

## 4. Motion Estimation

To challenge motion estimation in each environment, we developed a reliable and efficient method for switching an algorithm by a hybrid algorithm in order to compute the motion flow of the consecutive frames. This method was separated into two functions, which included a KLT tracker and an IMU-aided motion estimator.

### 4.1. A KLT Tracker

In case the absolute of $\omega_{z}^{cam}$ is smaller than 0.5 rad/s, then the rigid transformation is estimated by the corresponding set of the feature points. We use Good Feature to Track which is an efficient detector in real-time for calculating the optical flow. Feature points are detected by Harris corner detector, which uses the difference in intensity for a displacement of (*u*, *v*) in all directions, defined as follows:

(2) $$\begin{array}{cc} \varepsilon (u,v) & =\sum_{x,y}w(x,y){\left[I(x+u,y+v)-I(x,y)\right]}^{2}\\ & \approx \left[\begin{array}{cc} u & v \end{array}\right]Μ\left[\begin{array}{c} u\\ v \end{array}\right] \end{array}$$

where *I*(*x*, *y*) represents image pixels from the reference image and *I*(*x* + *u*, *y* + *v*) is the image pixels of the next image. *w*(*x*, *y*) is a Gaussian window function, which assigns weight to the surrounding pixel and *M* is the summary matrix derived from the gradient of the function in the specified neighborhood of a feature point. Moreover, the Harris corner detector score function as modified by Shi-Tomasi [29] is:

(3) $$R=\min(\lambda_{1},\lambda_{2})$$

where *λ*_1_ and *λ*_2_ are the eigenvalues of *M*. The result of *R* can divide to three cases: (1) if *λ*_1_ and *λ*_2_ are small which mean *R* is small also, then the region is flat; (2) if *λ*_1_ larger than *λ*_2_ then *R* is negative, so the region is an edge; (3) if *λ*_1_ and *λ*_2_ are large then *R* is large, and the region is corner. This improved method is called the Good Feature to Track.

**Figure 2:**
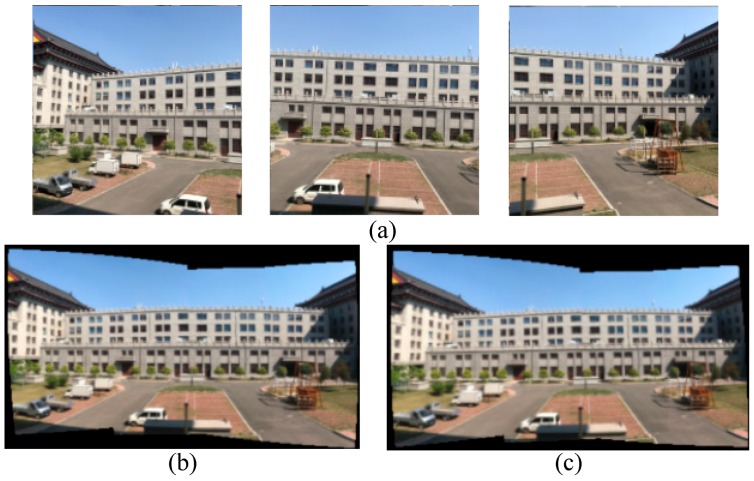
Examination of the feature points efficiency (**a**) the original images; (**b**) the panorama image with 200 feature points; and (**c**) the panorama image with 2000 feature points.

However, to run the algorithm in real-time, the feature points and their matching paired two consecutive frames essential during the computation time. We experimented the stitching panorama to confirm the efficiency of a certain number of feature points, which was sufficient to the homography matrix. Figure 2a shows the original of the images before stitching, Figure 2b,c illustrates the panorama images using 200 feature points and 2000 feature points, respectively. Those images were similar, but the large feature points overly computed the stitching image. Thus, our proposed method used less than 200 feature points to allow a reasonable estimate of the motion transformation.

The matching of the feature point between two consecutive frames needs to be approximated quickly. Therefore, we use the optical flow to match the detected corner. Optical flow is the pattern of apparent motion of image objects between the continuous frames due to the motion of the object or the camera. It represents the displacement of the 2D vector field (*dx*, *dy*) when a corner point is moving from the previous frame *I*(*x*, *y*, *t*) to the current one after *dt* time. Optical flow assumes the brightness does not change, then giving the following equation:

(4) I(x,y,t)=I(x+dx,y+dy,t+dt)

Equation (4) can be formulated in the simple form by removing the common term and dividing by *dt* after taking Taylor series approximation on the right-hand side. We obtained the image gradients *f_x_* and *f_y_* and the gradient along time *f_t_* to write the following equation:

(5) $$f_{x}u+f_{y}v+f_{t}=0$$

where

(6) $$f_{x}=\frac{\partial f}{\partial x};f_{y}=\frac{\partial f}{\partial y};u=\frac{dx}{dt};v=\frac{dy}{dt}$$

However, (5) have two unknowns (*u*, *v*) within one equation. Several researchers have proposed a method to solve this problem, but we used the Lucas–Kanade method, which is the standard method to estimate the optical flow. Lucas–Kanade solved this problem by taking the neighboring pixels with a 3 × 3 patch around the corner point, which assumed all the 9 points were of the same motion. Then, the two unknowns with right equations can be solved with the least square fit method as defined by:

(7) $$\left[\begin{array}{c} u\\ v \end{array}\right]={\left[\begin{array}{cc} \sum_{i}f_{x_{i}}^{2} & \sum_{i}f_{x_{i}}f_{y_{i}}\\ \sum_{i}f_{x_{i}}f_{y_{i}} & \sum_{i}f_{y_{i}}^{2} \end{array}\right]}^{-1}\left[\begin{array}{c} -\sum_{i}f_{x_{i}}f_{t_{i}}\\ -\sum_{i}f_{y_{i}}f_{t_{i}} \end{array}\right]$$

**Figure 3:**
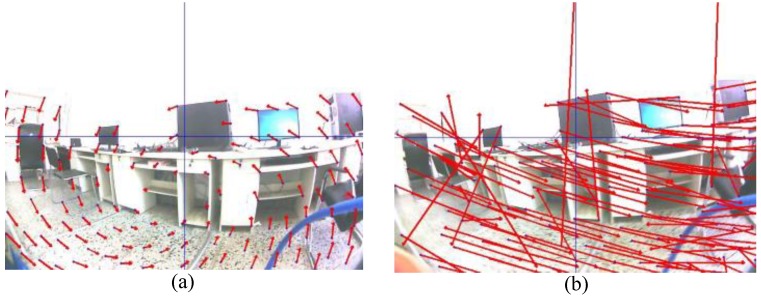
The motion vector from a Kanade–Lucus–Tomasi (KLT) tracker (**a**) case of low movement and (**b**) case of fast movement.

For example, Figure 3a,b shows the optical flow from a KLT feature tracker during a normal movement and a fast movement, respectively. The latter movement case being unordered, caused an inefficient estimate of the homography matrix. During large motion, the KLT feature tracker did fail. Thus, we used an IMU-aided motion estimator approach in the case of a large motion to reduce any motion vector error.

In summary, a KLT tracker has created the motion vectors from the feature points in the previous frame and the feature points that moving to a new location in the current frame. Those two sets of the feature points from KLT tracker are used to estimate the homography matrix of the rigid transformation to stabilize the image frames.

### 4.2. An IMU-Aided Motion Estimator

**Figure 4:**
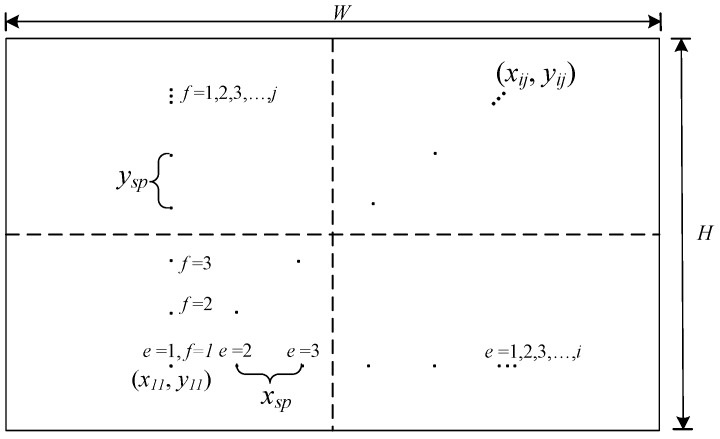
The reference points with a symmetrical pattern for IMU-aided motion estimator.

In the cases of absolute of $\omega_{z}^{cam}$ being more than 0.5 rad/s then the motion will be estimated by an IMU sensor. Firstly, we need to create the set of the reference point on the first image *I*_0_(*x_ij_*, *y_ij_*) as shown in Figure 4, by the symmetrically distributed point of (*i*, *j*). Size of (*i*, *j*) is calculated by (2*e* + 1) x(2*f* + 1), *e* and *f* = 1, 2, …, *n* where *e* and *f* should be between 5 and 30 to accurately approximate the motion with low computation load in the *x* and *y* directions, respectively.

All reference points of *I*_0_(*x_ij_*, *y_ij_*) are located around of the center of the image (*x*_0_, *y*_0_), which is determined by the equations below.

(8) $$\{\begin{array}{c} x_{ij}=x_{0}-x_{sp}(2e-1)/2+ix_{sp}\\ y_{ij}=y_{0}-y_{sp}(2f-1)/2+jy_{sp} \end{array}$$

where *x_sp_* and *y_sp_* are the pixel space between the reference points in the *x* and *y* directions, respectively. The motion point on the next frame *I_imu_*(*p*, *q*) can be calculated from *ω_cam_* on the *z*-axis in (1) which is given by:

(9) $$\{\begin{array}{c} p=(x_{ij}-H/2)\cos\varphi +(y_{ij}-W/2)\sin\varphi +H/2\\ q=-(x_{ij}-H/2)\sin\varphi +(y_{ij}-W/2)\cos\varphi +W/2 \end{array}$$

where *φ* equals to $\omega_{z}^{cam}$ divided by the frame rate of the input video, *H* and *W* are the height and width of the image, respectively. However, Li and Ren [16] performed the motion point with counteracting effect from the rotating motion in the *z*-axis, that is expressed as follows:

(10) $$\varphi =\{\begin{array}{ll} -8/F_{fps}, & \omega_{z}^{cam}<-6\\ -4/F_{fps}, & -6\leq \omega_{z}^{cam}\leq -2\\ 0, & 0.5\leq \left|\omega_{z}^{cam}\right|<2\\ 4/F_{fps}, & 2\leq \omega_{z}^{cam}\leq 6\\ 8/F_{fps}, & \omega_{z}^{cam}>6 \end{array}$$

**Figure 5:**
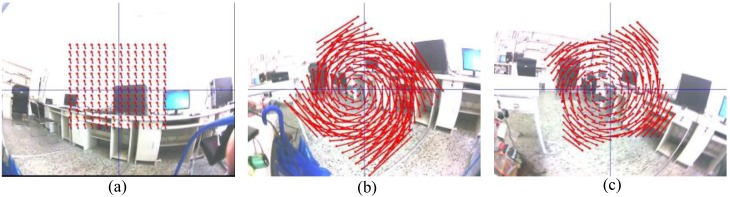
Motion vector with different rotational rates (**a**) $\omega_{z}^{cam}$ = −1.8 rad/s, (**b**) $\omega_{z}^{cam}$ = 6.58 rad/s and (**c**) $\omega_{z}^{cam}$ = −2.27 rad/s.

Therefore, from (9) and (10) can be generated by the motion vector from an IMU sensor. Direction and size of the motion vectors depend on the rotation measured. Figure 5 shows the motion vector that were calculated from set of points with different values of *φ*. For low rotation rate in Figure 5a, the pattern of the motion vector was similar in terms of direction and size. On the other hand, the pattern of the motion vector in fast rotation were in different direction and size, which was ordered, as illustrated in Figure 5b,c.

**Figure 6:**
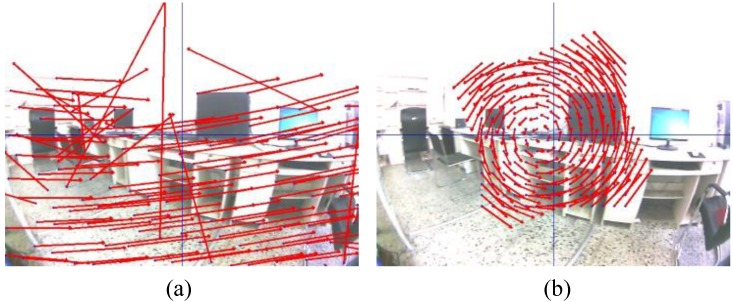
The motion flow at $\omega_{z}^{cam}$ = −2.82 rad/s (**a**) a KLT tracker and (**b**) an IMU-aided motion estimator.

The set of reference points and the new location points from an IMU sensor were applied to estimate the homography of a rigid transformation. The motion flow of two methods is compared the motion vector as shown in Figure 6, and the optical flow of a KLT tracker is complex for the fast rotation while the movement which is estimated from an IMU data is organized.

The approximated set point of the reference image (*S*_0_) and the current image (*S_n_*) in the previous step are used to define the motion model. The motion estimation approximates an affine transform [*A*|*t*] between those setpoints by finding a 2 × 2 matrix *A* and 2 × 1 vector *t*, which is formulated as shown below:

(11) $$\left[A^{*}|t^{*}\right]=\arg\;\min{\sum_{i}\left|\left|S_{0}[i]-AS_{n}{\left[i\right]}^{T}-t\right|\right|}^{2}$$

To solve [*A*|*t*] in (11), the matching pair required three pairs in a minimum to generate an affine transform. Thus, the motion estimation is found as an affine transform, that is applied to *I*(*x*, *y*) for mapping the warping image *I_warp_*(*x*’, *y*’).

(12) $$\left[\begin{array}{c} x^{\prime }\\ y^{\prime }\\ 1 \end{array}\right]=\left[\begin{array}{ccc} S\cos\theta & -S\sin\theta & t_{x}\\ S\sin\theta & S\cos\theta & t_{y}\\ 0 & 0 & 1 \end{array}\right]\left[\begin{array}{c} x\\ y\\ 1 \end{array}\right]$$

The degree of freedom (12) includes the scale (*S*), the rotation angle (*θ*) and the translation *t_x_* and *t_y_* in *x*- and *y*-axes, respectively. Thus, the total degree of freedom in (12) is four which is called similarity transformation. However, in this paper, we used rigid transformation to warp the stabilized frames, which gave the scale equal to 1 to reduce the store area for computing the scale vector. 

## 5. Motion Smoothing and Image Warping

To remove the noise from the estimated motion, we used Kalman filter to reduce the latter and obtained the smooth motion [30]. The Kalman filter approximated the next states by using the previous state [31], that suited the dynamic system of the consecutive frames. The predicted state of *x_k_* is given by:

(13) $$x_{k}=Fx_{k-1}+Bu_{k}+\omega_{k}$$

where *F* matrix is the state transition model in the previous state *x_k_*_−1_, *B* matrix is the control-input model, *u_k_* is the control vector, and *ω_k_* is the process noise in the Gaussian distribution. The state *z_k_* of the system state *x_k_* at time *k* is given by:

(14) $$z_{k}=H_{k}x_{k}+\nu_{k}$$

where *H_k_* and *v_k_* are the observation matrix and the observation noise, respectively. The filtered motion from the Kalman filter warped the stabilized frame with a smooth trajectory. Thus, the correction of rigid transformation can be found as:

(15) $$\left[\begin{array}{c} x_{sta}\\ y_{sta}\\ 1 \end{array}\right]=\left[\begin{array}{ccc} \cos(\theta -\hat{\theta }) & -\sin(\theta -\hat{\theta }) & t_{x}-{\hat{t}}_{x}\\ \sin(\theta -\hat{\theta }) & \cos(\theta -\hat{\theta }) & t_{y}-{\hat{t}}_{y}\\ 0 & 0 & 1 \end{array}\right]\left[\begin{array}{c} x^{\prime }\\ y^{\prime }\\ 1 \end{array}\right]$$

where ($\hat{\theta },\;{\hat{t}}_{x},$ and ${\hat{t}}_{y}$) is the filtered motion from the Kalman filter. The stabilized video is created with this correction motion and smoothness trajectory.

## 6. Multi-Threaded Approach for Video Stabilization

**Figure 7:**
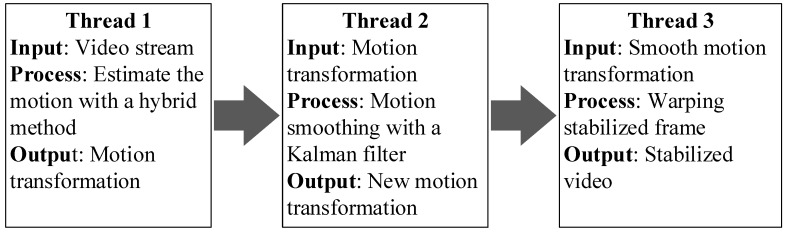
Multi-threaded approach for the video stabilization system.

The primary process of the stabilized video has three steps: motion estimation, motion smoothing, and image warping. For implement our algorithm in real-time, we managed the execution of each step algorithm with the multi-threaded approach. It can be processed into an array of commands in a single process, executed independently along with sharing the processing resources. To array process of multi-threaded approach in the video stabilization, we separated into three processes: thread1 for motion estimation, thread2 for motion smoothing and thread3 for image warping as shown in Figure 7.

The motion estimation thread inputted the video stream from the moving camera. A hybrid method switched the algorithm to estimate the motion, and it kept the estimated motion from the first 5th input frames before feeding to another process; all algorithms of the stabilized video were simultaneously processed after the 5th frame. Thus, from the 6th frame, the motion smoothing and image warping started the computation. Consequently, the accuracy motion in thread2 collected the probable motion in thread1 dynamically from the 2nd frame to the 5th frame.

The multi-threaded approach delayed in the starting process because thread2 and thread3 used the carried out data from thread1. However, the whole process continued until the last frame, and the threaded approach increased the frame rate as compared to the single-threaded approach demonstrated in the next section.

## 7. Experimental Results and Discussion

**Figure 8:**
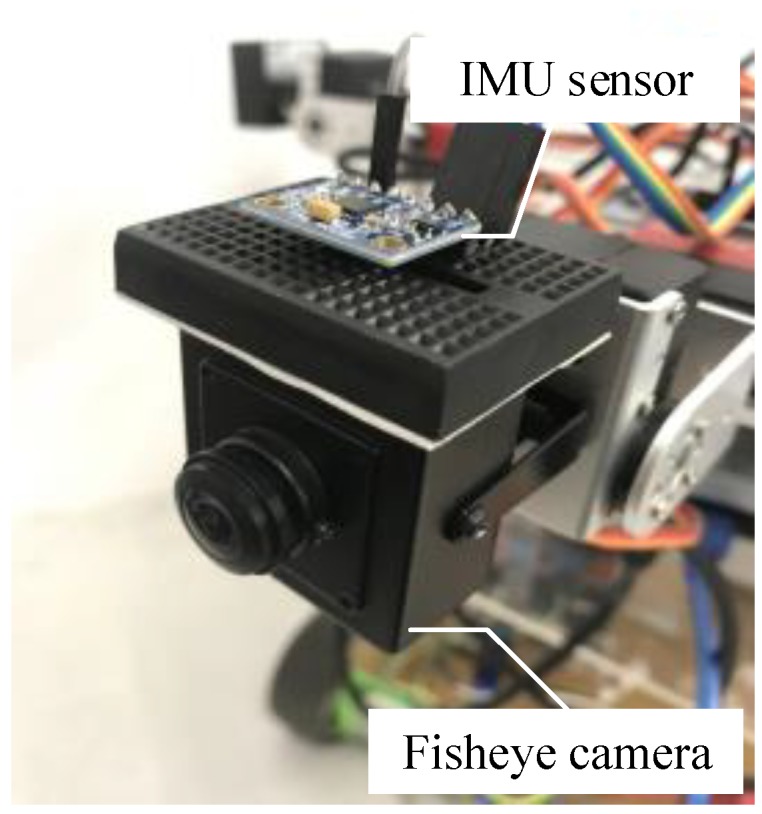
The experimental setup includes the fisheye camera and an IMU sensor as a GY-85 model.

In the experiment, we implemented our algorithm by using the ELP-4025 fisheye camera (produced by Ailipu Technology Co. Shenzhen, China) and an IMU as GY-85 model as shown in Figure 8. The GY-85 model consists of 3-axis accelerometer, 3-axis gyroscope, and 3-axis magnetometer. The fisheye camera and the IMU sensor were used because of being low-cost and also can be applied in the future development of surveillance applications in the mobile robot. The resolution of the captured video was 640 × 480 pixels. We developed our algorithm with C++ and also used the basic module from OpenCV.

Before stabilizing the video, the camera and the IMU sensor were calibrated. Firstly, we calibrated the camera with the chessboard by using the camera calibration module in OpenCV to determine the focal length. Secondly, we calibrated the gyroscope to prevent the drift problem by averaging the bias offset. The bias offset could also be determined by measuring the output signal of the gyroscope that reduced the noise in the original signal through the Kalman filter, as over a long period this sensor was sitting still. Then, we achieved the averaging of the bias offset in Equation (1) from 15 experiments with standard deviation (STD). The calibration data is shown Table 1. Next, *R^ci^* was estimated by using the measured data from the gyroscope in the IMU sensor and the optical flow (see detail [16]). In addition, the average of *R^ci^* from 15 experiments with the CC+LS13 method in [16] are shown in Table 1, which represents in term of quaternion.

**Figure 9:**
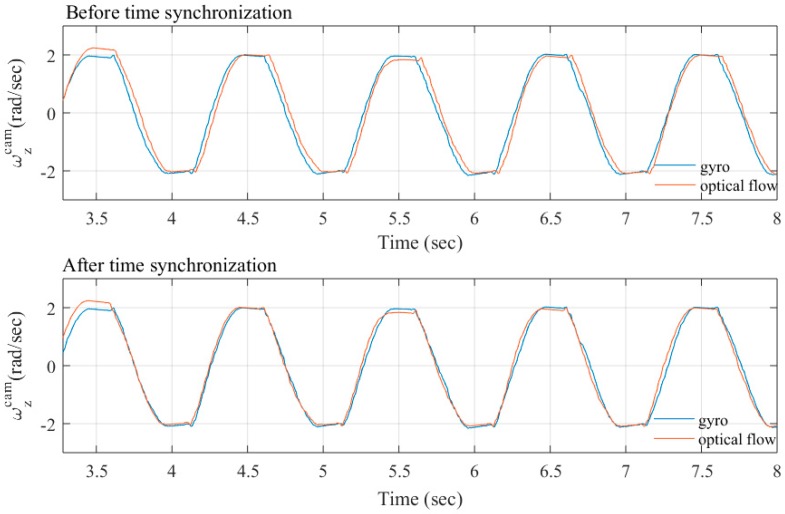
Time synchronization of the camera rotation from the gyroscope and optical flow.

Moreover, the camera and the IMU sensor are synchronized and operate between both of the sensors to accurately read data in time by a cross-correlation method. The temporal synchronization assumed the delay from the sensors which is constant [32]. This is a simple and easy way to define time lag between the two measurements. It can identify the offset time by applying the small sinusoidal signal required for moving the camera around the *z*-axis to measure $\omega_{z}^{cam}$. Then, comparison of the average magnitude of optical flow and gyro data is done as shown in Figure 9. The optical flow is estimated with the image interpolation algorithm [33] and the gyro data is calculated from Equation (1). The archived data from the optical flow and gyro data have the same phase included so the maximum phase lag between the two measured data represented by toff, equals to −0.035 s, as in our case.

Our proposed method was compared with a standalone of a KTL tracker and an IMU-aided motion estimator. To evaluate the performance of the stabilized video with reasonably acceptable results, we used the inter-frame transformation fidelity (ITF) [34] to represent the quality of the stabilized video in a single value by summarized the peak signal to noise ratio (PSNR), that is given by:

(16) $$\mathrm{ITF}=\frac{1}{N_{\max}-1}\sum_{k=1}^{N_{\max}-1}\mathrm{PSNR}$$

where *N*_max_ is the number of frames and PSNR is used to perform the effectiveness of the stabilization method, which is defined as:

(17) $$\mathrm{PSNR}(I_{n},I_{n+1})=10\log\frac{I_{\max}^{2}}{\mathrm{MSE}(I_{n},I_{n+1})}$$

where *I*_max_ is the maximum pixel intensity of the video frame, and MSE is the mean square error between two consecutive frames, *I_n_* and *I_n_*_+1_, which is calculating in every pixel of the continuous frames in the stabilized video, can be defined as:

(18) $$\mathrm{MSE}(I_{n},I_{n+1})=\frac{1}{NM}\sum_{j=1}^{N}\sum_{i=1}^{M}{\left(I_{n}(i,j)-I_{n+1}(i,j)\right)}^{2}$$

where *N* and *M* are the frame dimensions. The high of ITF and PSNR represents the good quality of the stabilized video.

**Figure 10:**
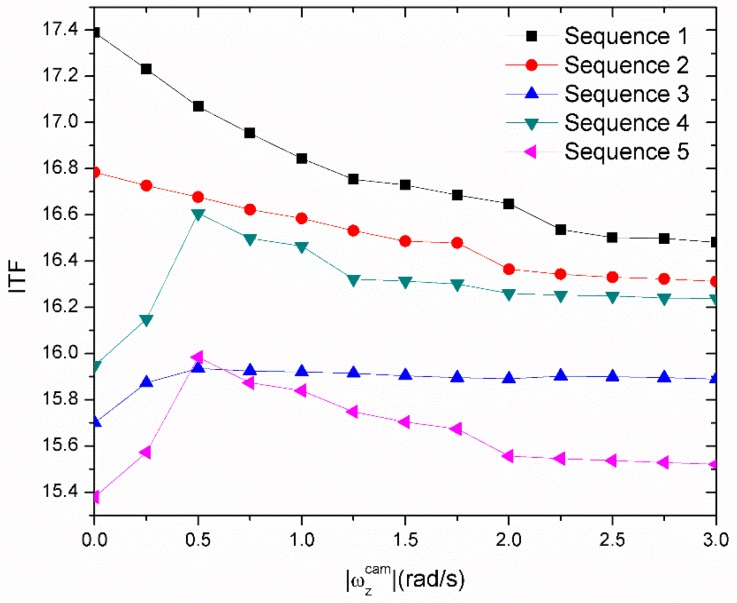
Inter-frame transformation fidelity (ITF) of video sequences with different switch threshold rotation speeds.

We used the IMU sensor data to optimize the switching threshold rotation speed. The switching threshold of different $\left|\omega_{z}^{cam}\right|$ values resulted in low ITF while using the high $\left|\omega_{z}^{cam}\right|$ to switch, as shown in Figure 10. Motion was not estimated at low rotation speeds during the high percentage case of motion estimation, as in Sequences 3–5. The highest IFT was obtained at $\left|\omega_{z}^{cam}\right|$ equaled to 0.5 rad/s. IFT decreased in Sequences 1 and 2 while the switching threshold changed to a higher value, because it was not sufficiently active in the high-percentage case of low $\left|\omega_{z}^{cam}\right|$.

**Figure 11:**
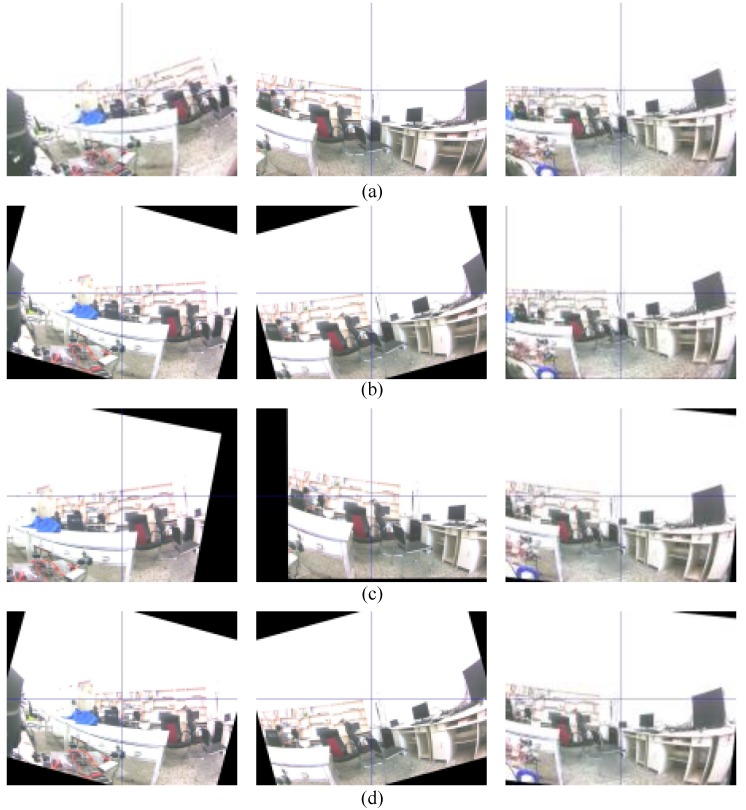
Sequence frame of the moving camera (**a**) is an original video (left: 48th, center: 168th frame, right: 227th frames); (**b**) Stabilized frame by an IMU-aided motion estimator; (**c**) Stabilized frame by a KLT tracker; and (**d**) Stabilized frame by a hybrid method.

Figure 11 shows the results of the stabilized video with other methods when the $\omega_{z}^{cam}$ of the 48th, 168th and 227th sequence frames were at −7.7 rad/s, 6.07 rad/s and −1.21 rad/s, respectively. The Kalman filter was warped with rigid transformation. The stable results of an IMU-aided motion estimator in the 48th and 168th frames were working better than the KLT tracker, especially in the case where it was fast rotated due to the KLT tracker failing to optimize the optical flow. Conversely, when the rotation was low in the 227th frame, a KLT tracker more efficient than the IMU-aided motion estimator due to the rotational signal being feeble. Thus, our improvement of an auto-switching algorithm included all advantages of both methods to illustrate the stabilized video as shown in Figure 11c, that was switching to an IMU-aided motion estimator in the 48th and 168th frame and was switching to the 227th frame regarding fast rotations and low rotations, respectively.

Moreover, we implemented five sequences with 300 frames for each sequence to demonstrate the ITF value of our algorithm when compared with the other method as shown in Table 2 and Table 3, which is fairly evaluated. It was clear to perform our algorithm switch to both methods depending on the motion behavior. Sequence 1 and Sequence 2 showed the ITF of our proposed method that resembled a KLT tracker due to the percentage of the estimation method with both method being similar, and an IMU-aided motion estimator was inefficient in case of low rotations. Hence, it means a KLT tracker is useful for low rotations. However, in the case of fast rotations more than the case of low rotations, e.g., Sequences 3–5, the standalone of the IMU-aided motion estimator was more for the ITF than that a KLT tracker. Moreover, in the case of fast rotations, the IFT of our proposed model is better than standalone KLT tracker and an IMU-aided motion estimator because our algorithm can conduct all rotational behaviors. The demos of the stabilized video is shown in the following link: https://youtu.be/ZS_PLFBBRAM.

**Figure 12:**
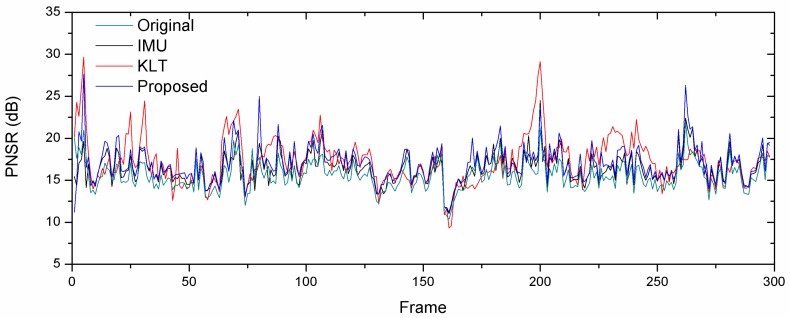
Comparison of PSNR with a different motion estimator.

In Figure 12, we represent the PSNR of Sequence 1 that was used to calculate the ITF, as shown in Table 3 demonstrates the stabilized efficiency. It can be seen that the value of an original video is lower than the stabilized video in different motion estimation methods. The PSNR of the KLT tracker is higher than other methods, as shown by the data in Table 3 showing the latter with a high ITF. But some of the consecutive frames with a KLT tracker are under the original video, which means the stabilized video in the subsequent frame is rough due to a KLT tracker failure. On the contrary, with our proposed method maintains the PSNR over the original video. It means the whole video of our proposed method achieved smoothness without any rough display on the video.

The maximum and average of MSE, are shown in Table 4 through comparison of the other methods. Most of the values from our proposed method was less than the standalone of a KLT tracker and an IMU-aided motion estimator, especially when the camera was moving with high rotation in the Sequences 3–5.

**Figure 13:**
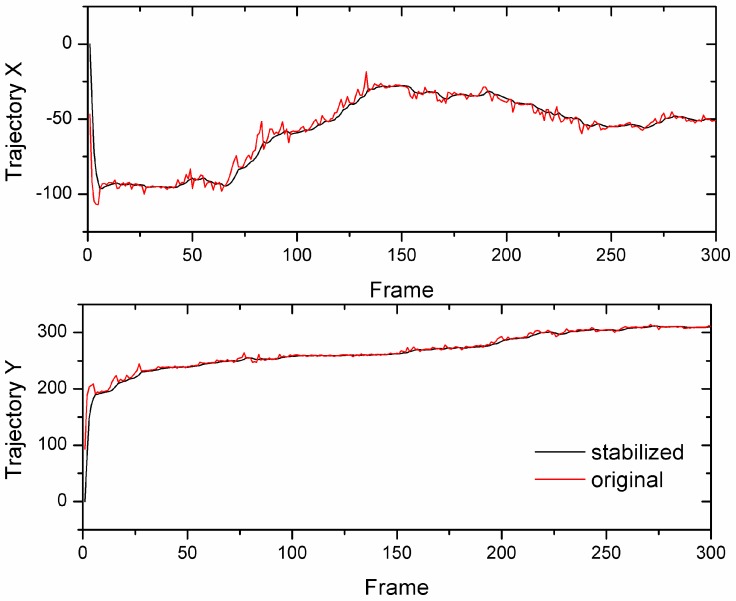
The smooth trajectory from Kalman filter in the video of Sequence 1.

The effect of the motion is illustrated in Figure 13, which showed our effective approach in smoothening the large motion in Sequence 1 from the Kalman filter. It improved the evenness over the originally measured motion by removing the undesired motion in both the *x* and *y* trajectories. Both offsets in the *x*- and *y*-trajectories of the stabilized video was smoother than the original one and that calculating in real-time.

Furthermore, we examined the execution of our algorithm in real-time with diverse threaded approaches. We implemented the algorithm on an Intel NUC Core i5-5250U at a frequency of 1.60 GHz, operating on ROS (Robot Operating System) in the Ubuntu platform. In the case of the single-threaded approach, we used the recorded rotational data from real-time, loading it offline for estimating the motion of high rotations with the IMU-aided motion estimator. Table 5 shows the comparison between single-threaded and multi-threaded approaches, respectively, showing an average frame rate for the multi-threaded approach to be 25.1 fps, which increased by 26.7%, compared to single-threaded approach.

The higher frame rate of the multi-threaded approach, more than the single-threaded approach shared the data with another thread suiting our proposed algorithm, described in real-time stabilization. The multi-threaded approach reduced the processing time to smoothen the trajectory because it could be calculated immediately with no waiting of the motion estimated data. Nevertheless, it has limited frame speed due to the firmware of the low-cost camera. Thus, this approach used less memory and low computational load to synchronize the threads of the stabilized video in real-time, which could be implemented in the high-level applications.

## 8. Conclusions

This paper proposed an auto-switching algorithm for motion estimation using IMU data. The KLT tracker and IMU-aided stabilization system were both used to approximate the motion. The proposed method effectively resolved unstable video in cases of fast rotation; it removed undesired motion at high efficiency compared to a standalone KLT tracker or an IMU-aided stabilization system. The proposed method did not yield superior results in the case of low rotations but performed similarly to the KLT tracker, which is commonly used for stabilizing videos. Likewise, the multi-threaded approach improved the real-time efficacy by processing the execution in the array. In the future, we plan to integrate our algorithm into an around-view system to further enhance its monitoring performance.

## Figures and Tables

**Table 1:** The camera and a gyroscope calibration result.

	*f* (*pixel*)	Gyro Bias Offset			Relative Orientation (*R^ci^*)				*t_off_*
		$b_{x}^{imu}$	$b_{y}^{imu}$	$b_{z}^{imu}$	$q_{0}$	$q_{1}$	$q_{2}$	$q_{3}$	
Average	326	0.234	−0.184	0.217	0.743	0.566	0.456	0.493	−0.035
STD	8.21	0.004	0.0011	0.007	0.028	0.019	0.012	0.009	0.033

**Table 2:** Percentage of the motion estimation cases.

Sequence	$\left|\omega_{z}^{cam}\right|<0.5$	$\left|\omega_{z}^{cam}\right|\geq 0.5$
Sequence 1	40.33	59.67
Sequence 2	47.67	52.33
Sequence 3	33.67	66.33
Sequence 4	29.33	70.67
Sequence 5	27.67	72.33

**Table 3:** Comparison of ITF by the different algorithm.

Sequence	Original ITF	Stabilized ITF			% Stabilized ITF		
		Proposed	KLT	IMU	Proposed	KLT	IMU
Sequence 1	15.618	17.070	17.391	16.491	9.303	11.358	5.591
Sequence 2	15.270	16.678	16.785	16.002	9.221	9.920	4.793
Sequence 3	14.777	15.936	15.700	15.887	7.847	6.247	7.513
Sequence 4	15.427	16.607	15.948	16.237	7.652	3.378	5.253
Sequence 5	14.796	15.984	15.379	15.518	8.024	3.935	4.877

**Table 4:** Error of two consecutive frames by different motion estimation method.

Sequence	Maximum				Average			
	Original	Proposed	KLT	IMU	Original	Proposed	KLT	IMU
Sequence 1	5999.0	5154.1	7565.6	4940.0	1948.2	1453.2	1443.2	1607.6
Sequence 2	4401.8	3593.1	4755.9	4198.3	2126.3	1592.9	1714.6	1822.3
Sequence 3	5077.1	4318.7	5228.0	4015.9	2362.7	1850.6	1841.9	2008.9
Sequence 4	4481.8	3922.6	6412.8	3669.5	2147.4	1680.7	1966.8	1754.0
Sequence 5	4317.5	3751.2	5564.7	4148.6	2356.9	1829.3	2185.5	1986.8

**Table 5:** Comparison of frame rate between single- and multi-threaded approach.

Sequence	Single-Threaded Approach [fps]	Multi-Threaded Approach [fps]
Sequence 1	20.3	25.6
Sequence 2	19.2	24.8
Sequence 3	19.7	25.1
Sequence 4	20.1	25.3
Sequence 5	19.9	24.9
